# A novel variant in *TAF1* affects gene expression and is associated with X-linked *TAF1* intellectual disability syndrome

**DOI:** 10.1042/NS20180141

**Published:** 2018-07-16

**Authors:** Sarah E. Hurst, Erika Liktor-Busa, Aubin Moutal, Sara Parker, Sydney Rice, Szabolcs Szelinger, Grant Senner, Michael F. Hammer, Laurel Johnstone, Keri Ramsey, Vinodh Narayanan, Samantha Perez-Miller, May Khanna, Heather Dahlin, Karen Lewis, David Craig, Edith H. Wang, Rajesh Khanna, Mark A. Nelson

**Affiliations:** 1 Department of Pathology, University of Arizona College of Medicine, Tucson, AZ 85724, U.S.A.; 2 Department of Pharmacology, University of Arizona College of Medicine, Tucson, AZ 85724, U.S.A.; 3 Department of Cellular and Molecular Medicine, University of Arizona College of Medicine, Tucson, AZ 85724, U.S.A.; 4 Department of Pediatrics, University of Arizona College of Medicine, Tucson, AZ 85724, U.S.A.; 5 Center for Rare Childhood Disorders, The Translational Genomics Research Institute (TGen), Phoenix, AZ 85004, U.S.A.; 6 Department of Family and Community Medicine, University of Arizona College of Medicine, Tucson, AZ 85724, U.S.A.; 7 Arizona Research Laboratories Division of Biotechnology, University of Arizona, Tucson, AZ 85721, U.S.A.; 8 Department of Pharmacology, University of Washington, School of Medicine Seattle, WA 98195, U.S.A.

**Keywords:** CACNA1G, cyclin D1, RNA sequencing, TAF-1, whole genome sequencing, X-chromosome inactivation

## Abstract

We investigated the genome of a 5-year-old male who presented with global developmental delay (motor, cognitive, and speech), hypotonia, possibly ataxia, and cerebellar hypoplasia of unknown origin. Whole genome sequencing (WGS) and mRNA sequencing (RNA-seq) were performed on a family having an affected proband, his unaffected parents, and maternal grandfather. To explore the molecular and functional consequences of the variant, we performed cell proliferation assays, quantitative real-time PCR (qRT-PCR) array, immunoblotting, calcium imaging, and neurite outgrowth experiments in SH-SY5Y neuroblastoma cells to compare the properties of the wild-type TATA-box-binding protein factor 1 (*TAF1*), deletion of *TAF1*, and *TAF1* variant p.Ser1600Gly samples. The whole genome data identified several gene variants. However, the genome sequence data failed to implicate a candidate gene as many of the variants were of unknown significance. By combining genome sequence data with transcriptomic data, a probable candidate variant, p.Ser1600Gly, emerged in *TAF1*. Moreover, the RNA-seq data revealed a 90:10 extremely skewed X-chromosome inactivation (XCI) in the mother. Our results showed that neuronal ion channel genes were differentially expressed between *TAF1* deletion and *TAF1* variant p.Ser1600Gly cells, when compared with their respective controls, and that the *TAF1* variant may impair neuronal differentiation and cell proliferation. Taken together, our data suggest that this novel variant in *TAF1* plays a key role in the development of a recently described X-linked syndrome, *TAF1* intellectual disability syndrome, and further extends our knowledge of a potential link between *TAF1* deficiency and defects in neuronal cell function.

## Introduction

Transcription of protein-encoding genes requires the concerted action of a large complex of transcription factors. The formation of the basic transcription machinery involves the assembly of a functional pre-initiation complex. Transcription factor II D (TFIID) is one of several general transcription factors that make up the RNA polymerase II pre-initiation complex. A minimal pre-initiation complex includes RNA polymerase II and six general transcription factors: TFIIA, TFIIB, TFIID, TFIIE, TFIIF, and TFIIH [1,2]. Moreover, TFIID is composed of the TATA box-binding protein (TBP) and several TBP-associated factors (TAFs) which are required for transcription from TATA-containing and TATA-less promoters. Thus, TFIID plays an important role in transcription initiation.

TATA-box-binding protein factor 1 (*TAF1*) (MIM: 313650) is the largest subunit of TFIID [1]. Bioinformatics analysis suggests that *TAF1* is a part of a 72-gene network involved in early brain development, and when mutated frequently causes genetic diseases [3]. In fact, a recognizable syndrome attributed to variants in *TAF1* has recently been described. X-linked *TAF1* intellectual disability (*TAF1*-ID) syndrome (MIM: 300966) occurs in males, and presents with global developmental delay, intellectual disability, characteristic facial dysmorphia, generalized hypotonia, and neurological abnormalities [4]. To our knowledge, approximately ten likely pathogenic variants have been reported for *TAF1*.

Next-generation sequencing (NGS) has revolutionized the investigation of pediatric diseases. The etiology and genetic basis of childhood disorders can be identified in approximately 25% of patients, where successful molecular diagnoses can often influence both patient management and treatment [5]. Integrated analysis of DNA and RNA from a patient may reveal genotype and phenotype correlation, and provide insight into the gene expression profile associated with a genetic condition, facilitating *in silico* predictions on the effects of genomic variants on gene expression, alternative splicing, exon usage, and gene fusions [6]. In the present study, whole genome sequencing (WGS) and mRNA sequencing was performed on a family having an affected male child who presented with global developmental delay (motor, cognitive, and speech), hypotonia, possibly ataxia, and cerebellar hypoplasia of unknown origin, and his unaffected parents and maternal grandfather. Based on the results, we report a novel *TAF1* variant c.4735A>G, p.Ser1600Gly (NM_004606) in a male patient. In addition, we provide data demonstrating that this *TAF1* variant identified appears to alter gene expression, cell proliferation, and neuronal differentiation.

## Materials and methods

### Enrollment of research participants

Specimen collection (i.e. whole blood samples) and genomic analysis was conducted at the University of Arizona and at the Translational Genomics Research Institute (TGen), as approved by their respective Institutional Review Boards. Written informed consent was obtained from the study participant, and the research was carried out in compliance with the Helsinki Declaration. As the patient was under 6 years of age at the time of enrollment, verbal assent was not required according to Western Institutional Review Board; written consent for the minor under the age of 18 years was obtained from the parents. The study protocol and consent procedure was approved by the Western Institutional Review Board (study number: 20120789).

### Clinical evaluation and diagnostics

Routine clinical diagnostic testing was performed on the patient including several genetic and metabolic panels. Cranial imaging was obtained by both MRI and computer tomography (CT).

### NGS analysis

The QIAamp DNA Blood Maxi Kit, Qiagen, was used to isolate DNA from 8 to 10 ml of whole blood from the patient’s mother, father, and the proband as previously described [7]. DNA libraries were prepared using the NEBNext DNA Sample Prep Master Mix Set (New England Biolabs, Ipswich, MA). For each sample library preparation, 1 μg of high molecular weight genomic DNA was fragmented using the Covaris S2 system. Fragmented samples were end-repaired using T4 DNA polymerase, DNA polymerase I Klenow fragment, and T4 polynucleotide kinase. Samples were next adenylated using Klenow fragment 3′–5′ exo minus enzyme, ligated with Illumina adapters, size selected at 350–450 bp, and PCR amplified using Phusion High-Fidelity PCR Master Mix w/HF buffer (New England Biolabs). The DNA libraries were clustered on to flow cells using Illumina’s cBot and HiSeq Paired End Cluster Generation kits as per manufacturer protocol (Illumina, San Diego, CA). NGS of the mother, father, and the proband were carried out using the Illumina HiSeq 2000 platform using the v1.5 chemistry reagents and flow cells. The samples was sequenced on the Illumina HiSeq 2000 platform using the v3 chemistry. The total length of each paired end sequencing run was 200 cycles. Multiple runs were performed to generate a minimum of 25× mean coverage on each sample after all post processing. Readings were aligned to the human reference genome (build 36) using Illumina’s ELAND2 pipeline, followed by somatic analysis to identify single-nucleotide variants (SNVs), indels, and copy number variants as described previously [8,9].

WGS variant analysis was performed as previously described by Veeramah et al. [9] with some modifications. Annotation was performed using SnpEff 3.2a [10] based on Hg19, and custom software tools were used to gain insight into the potential inheritance pattern of variants of interest (i.e. whether the variants could be associated with an autosomal dominant, autosomal recessive, or X-linked inheritance pattern). After annotation, the Ensembl Genes database (Ensembl v66) was used for gene annotation, variant classification, and to assess nucleotide and protein changes, while OMIM and ClinVar were used for disease and/or phenotype classification. At last, known variant databases (1000 Genomes, 6500 Exomes, dbSNP RSID, and Exome Aggregation Consortium (ExAC)) were searched to identify single nucleotide polymorphisms (SNP) and their frequency of occurrence, if available. Protein conservation and the mutational impact was assessed using several prediction software programs including CADD, PolyPhen2, SIFT, Mutation Taster, and PhyloP. A summary of the WGS statistics is listed in Supplementary Table S1.

### RNA sequencing

Whole blood samples were collected from the mother and the affected proband into PAXgene tubes (Qiagen). Ten nanograms of total RNA, isolated from whole blood samples from the unaffected mother and the affected proband, were used to generate separate whole transcriptome libraries using the Nugen Ovation RNA-Seq System v2 (San Carlos, CA) and Illumina’s TruSeq RNA Sample Preparation Kit. An equimolar pool of all four barcoded libraries was clustered on the cBot using the TruSeq PE Cluster Kit v3 and sequenced on the HiSeq 2000 platform. Here, filtered reads were mapped to Hg19/GRC37 with TopHat2 (v2.0.8) [11]. Subsequently, Cufflinks was used, which uses the aligned reads to assemble them into transcripts, estimates their abundance, and tests for differential expression [12,13]. For estimation of X-chromosome inactivation (XCI), ratio reads were aligned to human reference genome GRCh37.62 using TopHat2 [11].

### Validation by Sanger sequencing

The presence of the p.Ser1600Gly variant was corroborated by Sanger sequencing. Given the position of the variant, the PCR primers were designed to amplify a portion of the second bromodomain of the *TAF1* gene. Sanger sequencing was performed using an Applied Biosystems Sanger Sequencing Kit (Thermo Fisher Scientific). DNA sequencing was run on an Applied Biosystems 3730XL DNA Analyzer (Foster City, CA). The user can expect up to 600 bases of reliable sequence per read in one direction. The sequencing data were loaded into the ABI Sequence Scanner Software v1.0 for further analysis and genotype calling. All sequence traces were also manually reviewed to ensure the reliability of the genotype calls.

### Cell culture

SH-SY5Y human neuroblastoma cells were grown in Dulbecco’s modified Eagle’s medium: nutrient mixture F-12 (DMEM/F-12) (HyClone, South Logan, UT) medium containing 1% non-essential amino acids (Caisson, Smithfield, UT), 1% sodium pyruvate (Gibco, Gaithersburg, MD), and 1% penicillin/streptomycin (Gibco, Gaithersburg, MD) supplemented with 10% FBS (VWR, Denver, CO), in a 95% air/5% CO_2_ humidified incubator at 37°C. Cells were grown to confluency (80%), and the same passage number was used for all the experiments.

### *TAF1*-specific siRNA transfection

SH-SY5Y cells were seeded at 3.0 × 10^5^ cells/well in a poly-d-lysine coated six-well plate and maintained for 24 h in antibiotic-free DMEM/F-12. *TAF1* expression in SH-SY5Y cells was altered by transfecting cells with a commercially available *TAF1*-specific siRNA duplex (SR304693A) at a concentration of 10 nM, and a scrambled non-targetting control duplex (SR30004 NC1 Ctrl) also at 10 nM (Origene, Rockville, MD). Complete DMEM/F-12 was added after 6 h to ensure cell health. Cells were collected after 24 h post-transfection and used for subsequent experiments.

### Site-directed mutagenesis

The proband’s specific variant, p.Ser1600Gly, was introduced into full-length human *TAF1* cDNA in a pCS2HA expression plasmid [pCS2HA was a gift from Peter Klein (Addgene plasmid # 16330, Cambridge, MA)] by PCR using CloneAMP HiFi PCR Premix (Clontech, Mountain View, CA), 50 ng template DNA, and 0.25 mM each of the following primers: 5′-TCT-GGC-CAA-CGG-TGT-TAA-GTA-TAA-TGG-3′ and 5′-CCA-TTA-TAC-TTA-ACA-CCG-TTG-GCC-AGA-3′. After 20 cycles, PCR products were digested with DpnI, transformed into bacteria, and the variant identified by DNA sequencing. This plasmid as well as a *TAF1* wild-type plasmid were used in all subsequent transfection experiments excluding the calcium imaging.

For calcium imaging experiments, the *TAF1* variant p.Ser1600Gly was introduced into full-length human *TAF1* cDNA in a pCIG3 expression plasmid [pCMV-internal ribosome entry site (IRES)-GFP version 3 was a gift from Felicia Goodrum (Addgene plasmid # 78264, Cambridge, MA)]. To do so, NheI and BamHI restriction enzyme sites were added to the 5′ and 3′ ends, respectively, of human *TAF1* cDNA by PCR using Phusion polymerase (Thermo Fisher Scientific, Waltham, MA). The resulting product was then subcloned in front of an IRES-GFP sequence in the expression vector pCIG3. The *TAF1* variant p.Ser1600Gly variant was introduced by site-directed mutagenesis using Phusion polymerase with the following primers: 5′-TGG-CCA-ACG-TGT-TAA-GTA-TAA-TGG-ACC-3′ and 5′-CTT-AAC-ACG-TTG-GCC-AGA-ATA-AGG-3′. This plasmid as well as a *TAF1* wild-type were used for calcium imaging.

### *TAF1* wild-type and variant plasmid transfection

All the SH-SY5Y cells were treated according to the manufacturer’s instructions using siTrans (Origene) transfection reagent; the amount of DNA used was 360 ng. Complete DMEM/F-12 was added after 6 h to ensure cell health. Cells were collected after 24 h post-transfection and used for subsequent experiments.

### Cell proliferation assay

Cell proliferation was measured using a Quick Cell Proliferation Assay Kit (Abcam, Cambridge, MA). Following the manufacturer’s instructions, cells were seeded on to a 96-well plate (2.5 × 10^4^ cells/well). Once confluent (70%), the cells were transfected with *TAF1* plasmids. After 48 h post-transfection, WST-1/ECS solution was added to each well, and incubated for 2 h under standard culture conditions. After 2 h, the absorbance was measured at 420–480 nm (BioTeK Synergy HT, Winooski, VT). The percentage of cell proliferation was calculated, and results presented relative to control (100%).

### Cell differentiation and Sholl analysis

SH-SY5Y cells were seeded at 3.0 × 10^5^ cells/well in a six-well plate, and maintained for 24 h in antibiotic-free DMEM/F-12. Six hours after *TAF1* plasmid transfection, the medium was replaced with differentiation medium containing DMEM/F-12 and a cocktail of IGF-1, BDNF, NGF, and RA. The cells were monitored everyday and images were taken. The differentiation medium was replaced every 3 days until cells were fully differentiated, which took approximately 7 days. Upon completion, neurite outgrowth was evaluated. Phase-contrast images (20× magnification) were obtained from each well on an EVOS FL inverted microscope (Life Technologies, Carlsbad, CA) equipped with a CCD camera. The experimenter, blinded to the treatment, selected fields randomly. Digitized images of the cells were collected, and morphometric analysis was performed using ImageJ software. Neurite outgrowth was determined by measuring the length of neurites, and cells were considered as neurite bearing if the length of at least one neurite was longer than 50 µm.

### RNA isolation, cDNA synthesis, and semi-quantitative RT-PCR analysis

After 24 h post-transfection with either siRNA or plasmid, RNA was extracted and reverse transcribed to cDNA. *GAPDH* (housekeeping gene (HKG)) and *TAF1* gene mRNA expression was analyzed using quantitative real-time PCR (qRT-PCR). The percentage of *TAF1* depletion was calculated respective to the expression of the matched non-targetting scrambled control sample. Total RNAs were isolated from each sample using RNeasy Mini RNA kit (Qiagen, Valencia, CA), according to the manufacturer’s instructions. Single-stranded cDNA was synthesized using the High Capacity cDNA Reverse Transcription Kit (Applied Biosystems). To perform reverse transcription, the thermal cycler was programmed with the following conditions; Step 1: 25°C for 10 min, Step 2: 37°C for 120 min, and Step 3: 85°C for 5 min. The amplified cDNA was then quantitated on a Nanodrop (Thermo Fisher Scientific), and stored until further use at −20°C. Semi-quantitative RT-PCR was performed using the 96-Well RT^2^ Profiler™ Neuronal Ion Channels PCR array (Qiagen) as follows: after appropriate dilution, the cDNA template was added to the reaction mixture, and equal amounts were added to each well of the array, containing gene-specific primers. The experiment was performed on the Bio-Rad CFX96 (Hercules, CA) using the following thermal profile: segment 1 – 1 cycle: 95°C for 10 min, segment 2 – 40 cycles: 95°C for 30 s and 60°C for 1 min. The data were analyzed by SA Bioscience’s PCR Array Data Analysis Web Portal. To validate RT-PCR array results, amplified cDNA from samples were added to the RT^2^ SYBR Green Master Mix (Qiagen) along with gene specific primers (Supplementary Table S2) and RT-PCR was performed as described above. Each array contained three separate HKGs (*RPLP0, GAPDH*, and *ACTB*) that were used for normalization of the sample data. Normalization to the HKGs was performed by calculating the Δ*C*_t_ for each gene of interest (GOI) in the plate (*C*_t_ value of GOI − *C*_t_ value of HKG). Fold change is the normalized gene expression (2∧-Δ*C*_t_) of the *TAF1*-silenced SH-SY5Y cells minus the normalized gene expression (2∧-Δ*C*_t_) of the scrambled control SH-SY5Y cells.

A list of the genes encoding for different subunits of the ion channels is given in Supplementary Table S3.

### Preparation of cellular extracts, immunoblotting, and densitometry analysis

Total cell lysates were isolated using a commercial RIPA buffer according to standard methods. Briefly, cells were washed twice with 1× PBS and lysed in RIPA buffer containing a protease inhibitor cocktail at 4°C for 30 min. Lysates were centrifuged at 16000  ***g*** for 20 min at 4°C. Protein estimation was performed using the BCA kit (Thermo Fisher Scientific) according to the manufacturer’s instructions. Immunoblot analysis was carried out according to standard procedures, and 25−50 μg of protein lysates were resolved on gradient SDS/PAGE (4–15% gel) using 2× Laemmli sample buffer; cell lysates were denatured by heating before being applied to SDS/PAGE gel. After electrophoresis, proteins were transferred on to PDVF membranes, blocked for 1 h in blocking solution (5% dry milk in TBST buffer), and incubated with specific primary antibodies overnight at 4°C. Primary antibodies were detected with HRP-conjugated secondary antibodies, and antibody–protein complexes were developed using Clarity Western ECL Substrate (Bio-Rad) and visualized using a C-Digit imaging system (LI-COR Biosciences, Lincoln, NE). Results are expressed as the ratio of target protein expression to that of an internal loading control, β-ACTIN. Relative densitometry analyses of the immunoblots were determined using ImageJ analysis software. By giving an arbitrary value of 1.0 to the respective control sample (β-ACTIN) of each experiment, a ratio of relative density was calculated for each protein of interest.

### Calcium imaging

The *TAF1* plasmid transfected SH-SY5Y cells were loaded at 37°C with a 3 μM Fura-2AM (Thermo Fisher Scientific) stock solution prepared at 1 mM in DMSO and 0.02% pluronic acid (Thermo Fisher Scientific) for 30 min (*K*_d_ = 25 μM, λ_ex_: 340, 380 nm/λ_emi_: 512 nm) to follow changes in intracellular calcium ([Ca^2+^]_c_) in Tyrode’s solution (at approximately 310 mOsm) (119 mM NaCl, 2.5 mM KCl, 2 mM MgCl_2_, 2 mM CaCl_2_, 25 mM HEPES, and 30 mM glucose; pH 7.4). The solution was supplemented with 500 nM tetrodotoxin (TTX, voltage-gated Na^+^ channel inhibitor) and 1 μM nifedipine (L-type voltage-gated Ca^2+^ channel inhibitor). All calcium imaging experiments were performed at room temperature (approximately 23°C). Baseline was acquired for 1 min followed by stimulation (15 s) with an excitatory solution (at approximately 310 mOsm) (32 mM NaCl, 90 mM KCl, 2 mM MgCl_2_, 2 mM CaCl_2_, 25 mM HEPES, and 30 mM glucose; pH 7.4). Fluorescence imaging was performed with an inverted microscope, Nikon Eclipse T*i*-U, using objective Nikon S Plan Fluor ELWD 20× 0.45 (Melville, NY) and a Photometrics cooled CCD camera CoolSNAP ES^2^ (Roper Scientific, Tucson, AZ) controlled by NIS Elements software (version 4.20). The excitation light was delivered by a Lambda-LS system, and the excitation filters (340 ± 5 and 380 ± 7 nm) were controlled by Lambda 10-2 optical filter change (Sutter Instruments, Novato, CA). Fluorescence was recorded through a 505-nm dichroic mirror at 535 ± 25 nm. To minimize photobleaching and phototoxicity, the images were taken every 10 s during the time-course of the experiment using a minimal exposure time that provided quality images. The changes in [Ca^2+^]_c_ were monitored by following the ratio of F_340_/F_380_, calculated after subtracting the background from both channels.

### Computer modeling

To predict how the TAF1 variant might influence protein structure, we examined the published X-ray crystal structure of the tandem bromodomain [14]. The co-ordinates from 3UV5.pdb were used to model the p.Ser1600Gly variant using the mutation wizard in PyMol 1.8 (https://www.pymol.org/citing).

### Statistical analysis

For all experiments, we performed statistical calculations in Microsoft Excel and GraphPad Prism6. One-way, repeated-measure ANOVA with Bonferroni adjustment was used to compare differences between multiple groups. Post hoc analysis was conducted if warranted; Tukey tests (95%). The probability of significant differences between two groups was determined using Student’s *t* test, and the *P*-values were adjusted using Benjamini–Hochberg principles for correcting false discovery rate. The data were expressed as mean ± S.D. of three independent experiments performed in triplicate. A *P*-value of 0.05 was considered to indicate a statistically significant difference.

## Results

### Clinical evaluations and phenotypic presentation

**Figure 1:**
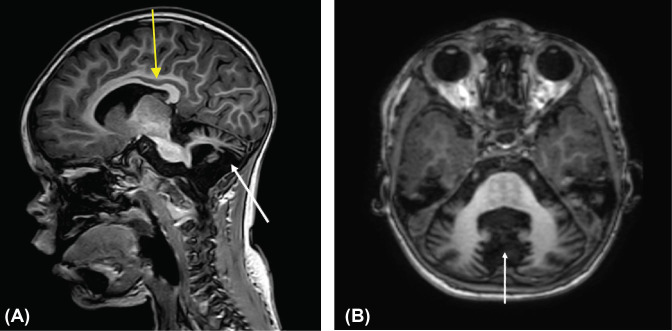
Twenty-one month old child with cerebellar hypoplasia. (**A**) Mid-sagittal T1-weighted image showing thinning of corpus callosum (yellow arrow) and decreased cerebellar volume (white arrow). (**B**) Axial T1-weighted image showing decreased volume of the cerebellar lobes (white arrow). With cerebellar hypoplasia, hemispheres are normal, but patients have small brain stems, particularly the pons and medulla. Other structural brain abnormalities may include thinning or absence of the corpus callosumaand communicating hydrocephalus.

Imaging and electroencephalogram (EEG) studies were conducted on the patient at 11 and 21 months of age to aid in condition diagnosis. At the age of 11 months, 24 h brain EEG (Barrows Neurological Institute, Phoenix, AZ) showed no seizure activity and no suspected seizure *foci*, with slight slowing of general cortical wave activity possibly indicative of cortical dysfunction. Cranial CT and MRI showed no synostosis or gross abnormalities of brain architecture, but revealed cerebellar hypoplasia and possibly delayed cortical myelination. Plain X-ray films showed no abnormalities of long-bone architecture or other orthopedic findings and an MRI of the spine appeared normal. A follow-up MRI at 21 months showed a marked loss of cerebellar volume (Figure 1) as well as enlargement of the fourth ventricle and cerebrospinal fluid (CSF) spaces in the *posterior fossa* with mild enlargement of the third ventricle. Interestingly, the scan suggested appropriate cortical myelination for the child’s age. However, the observed enlargement of the fourth ventricle and CSF spaces suggested some associated atrophy of the *pons* and middle cerebellar peduncle. Morphology of the patient’s spine at 21 months of age appeared normal.

Now at the age of 5 years, the patient continues to have delays in cognitive, gross motor, and fine motor skills, as well as developmental apraxia of speech. Moreover, the patient has postural instability, and delayed or absent protective reactions. The child also appears to have improving mild hypotonia, as clinical strength is generally good although weaker than normal in upper extremities. The child is generally happy, highly sociable seeking to connect with people, and has normal hearing. The child does display stereotypical neurological impairments, such as hand flapping/gesturing. The most recent MRI scan revealed no progression in the loss of cerebellar volume, but cerebellar hypoplasia as well as increased extra axial fluid is still present. There also appears to be some mild ventriculomegaly and delayed myelination. In regard to the affected proband’s phenotype, the affected male child has a broad forehead, anteverted nares, brachydactyly, thickened earlobes, minor myopia, supernumery nipples, thin lips, and up-slanted pupil fissures. Several of these clinical features are similar with probands affected with *TAF1*-ID syndrome [4] (Table 1).

**Table 1:** Summary of clinical features of the affected proband (11) identified in the present study and those previously reported probands (1–10)

		Proband										
Features	HPO	1	2	3	4	5	6	7	8	9	10	11
Sex		Male	Male	Male	Male	Male	Male	Male	Male	Male	Male	Male
Age (years)		15	13	5	6	22	11	UNK	UNK	UNK	UNK	4
TAF1 Variant (hg19)		p.Ile1337Thr	p.Ile1337Thr	p.Cys807Arg	p.Arg1246Trp	p.Asn1517His	p.Arg1431His	p.Met21Leu	p.Glu1428Pro	p.Asn493Asp	p.Arg1190Cys	p.Ser1600Gly
Inheritance pattern		Maternal	Maternal	*De novo*	*De novo*	*De novo*	*De novo*	Maternal	Maternal	Maternal	Maternal	Maternal
Delayed gross motor development	HP:002194	+	+	+	++	+	+	UNK	UNK	UNK	UNK	+
Delayed speech and language development	HP:0000750	+	+	+	++	+	+	UNK	UNK	UNK	UNK	+
Intellectual disability	HP:0001249	+	+	+	+	+	+	+	+	+	+	+
Prominent supraorbital ridges	HP:0000336	+	+	-	+	+	-	UNK	UNK	UNK	UNK	+
Prominent forehead	HP:0011220	+	+	+	-	-	+	UNK	UNK	UNK	UNK	+
Long philtrum	HP:0000343	+	+	-	+	-	+	UNK	UNK	UNK	UNK	+
Thickened helices	HP:0009748	+	+	-	-	+	+	UNK	UNK	UNK	UNK	+
Macrotia	HP:0000400	+	+	+	+	+	+	UNK	UNK	UNK	UNK	+
Broad upturned nose	HP:0000463	+	+	+	+	-	+	UNK	UNK	UNK	UNK	+
Bulbous nasal tip	HP:0000414	+	+	+	+	-	+	UNK	UNK	+	+	+
Myopia	HP:0000545	-	-	+	+	+	-	UNK	UNK	UNK	UNK	+
Hypoplasia of the cerebellar vermis	HP:0001320	+	+	++	-	-	-	UNK	UNK	UNK	UNK	+
Hypoplasia of the corpus callosum	HP:0002079	+	+	+	UNK	+	UNK	UNK	UNK	UNK	UNK	+
Hypotonia	HP:0001290	+	+	+	+	+	-	UNK	UNK	UNK	UNK	+
Hyporeflexia	HP:0001315	+	+	+	+	+	+	UNK	UNK	UNK	UNK	+
Gait abnormalities	HP:0001288	+	+	+	-	+	-	UNK	UNK	UNK	UNK	+
Balance problems	HP:0002141	+	+	-	+	+	-	UNK	UNK	UNK	UNK	+
Postural instability	HP:0001251	+	+	-	-	+	-	UNK	UNK	UNK	UNK	+
Bradykinesia	HP:0002067	-	-	-	-	+	-	UNK	UNK	UNK	UNK	+
Short digits	HP:0011927	-	-	+	-	-	+	UNK	UNK	UNK	UNK	+
Autistic behaviors	HP:0000729	+	+	+	-	+	+	UNK	UNK	UNK	UNK	-
Recurrent hand flapping	HP:0100023	UNK	UNK	UNK	UNK	UNK	UNK	UNK	UNK	UNK	UNK	+

‘+’ indicates mild or simple presence of the phenotype; ‘++’ indicates a more pronounced presence of the phenotype; ‘–’ indicates an absent feature. Abbreviation: UNK, unknown.

TAF1 variants in probands (1–6) were previously reported by O’Rawe et al. [4]. TAF1 variants in proband 7 and 8 were reported by Niranjan et al. [15]. The TAF1 variants’ probands 9 and 10 were reported by Hu et al. [16]. The TAF1 variant in proband 11 was reported by the present study.

### NGS analysis

WGS was performed on the child, the unaffected mother, and the father. For the *de novo* model, we found eight coding variants of sufficient quality (absence of multiple Mendelian inheritance errors in the gene and novel or <3% in population databases) for manual review. In the case of the *de novo* model we also filtered out variants with VAF <30% in the proband or >10% in either parent. After PCR and Sanger sequencing, all eight candidates were eliminated. We also examined compound heterozygous and simple recessive inheritance models, which revealed that 24 and 52 variants that passed our quality standards described above. None of these candidates appeared to be associated with the patient phenotype based on literature, OMIM, and ClinVar database searches. For the eight X-linked variants that passed our quality filters, two variants of unknown significance (VUS) in the *MECP2* and *TAF1* genes were identified. The *MECP2* variant was ruled out due to benign pathogenicity reports, clinical experience (i.e. ClinVar), and the child’s phenotype. Finally, we found that none of the other variants (*n*=268) in our set of candidate genes that play a role in neurodevelopment were predicted to be damaging and/or unique to the proband. A similar analysis of 406 variants within 5163775 phastCons conserved elements did not produce candidates in genes or linked regions known to be related to the proband’s phenotype. As such, the *TAF1* variant was left as the sole candidate, provided the mother showed X-chromosome skewing.

**Figure 2:**
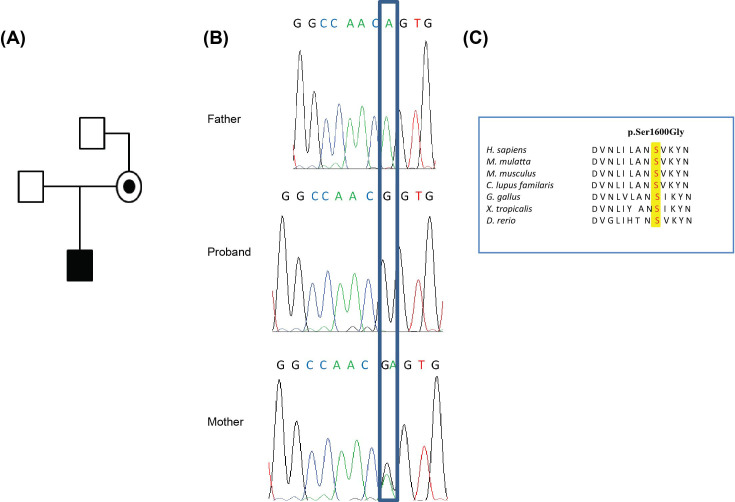
Variant in *TAF1* identified in the affected proband (**A**) Pedigree of the family. (**B**) Sanger sequencing chromatogram showing the segregation analysis of the p.Ser1600Gly variant identified in *TAF1*. (**C**) Peptide alignments showing the conservation of the affected amino acid across different species.

The sequencing of the child’s genome identified a VUS c.4735A>G, p.Ser1600Gly in the *TAF1* gene. Segregation analysis showed that the mother was a heterozygous carrier for this gene variant, while the father was wild-type (Figure 2A,B). In regard to *TAF1*, the variant had not been previously identified, and had not been observed in control populations including the NHLBI Exome Sequencing Project and the 1000 Genomes project. The amino acid Ser^1600^ in *TAF1* is conserved from *Danio rerio* to *Homo sapiens* (Figure 2C). Exome sequencing was performed on the maternal grandfather (see Supplementary data for details). The *TAF1* variant was not present in grandfather (data not shown). Other members (i.e. the maternal grandmother) of the family were unavailable for sequencing. Thus, we could not confirm if the mother inherited this *TAF1* variant or if it occurred *de novo.*

A skewed XCI ratio (i.e. 90:10) is suggestive of non-random inactivation, which can play an important role in X-linked genetic conditions. We observed an X-inactivation ratio of 96:4 in the mother of the proband, which is consistent with a highly skewed X-inactivation pattern (Supplementary Figure S1). Phased allele expression analysis confirmed extreme skewing in the mother and also showed preferential expression of the wild-type allele is mediated by preferential X-inactivation. Moreover, the phased allele specific expression shows the direction of skewing and that allele specific expression covers the entire X-chromosome (Supplementary Figure S2). These data implicated the *TAF1* p.Ser1600Gly variant as the most likely causative or contributing gene to the child’s phenotype.

### Computer modeling and bioinformatics analysis

**Figure 3:**
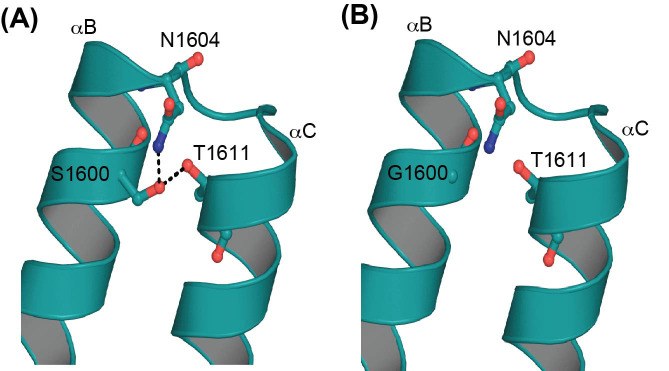
Molecular model of the second bromodomain of TAF1 (**A**) The S1600 side chain makes H-bond contacts with N1604 on the next turn of αB and with T1611 on the adjacent helix αC. The S1600, N1604, and T1611 are conserved. (**B**) Replacement of the serine with glycine is predicted to disrupt hydrogen bonding with N1601 and T1611.

The *TAF1* variant p.Ser1600Gly we identified is located in a conserved position within the second bromodomain. The bromodomains of TAF1 are composed of four α helices that form a hydrophobic pocket that recognizes an acetyllysine such as those on the N-terminal tails of histones. To predict how the *TAF1* variant might influence protein structure, we examined the published X-ray crystal structure of the tandem bromodomain [14]. In the native state, S1600 makes two hydrogen bonding contacts, one with conserved N1604 within the same helix, and the second with T1611 in the neighboring helix (Figure 3). The N1604 amino acid has been shown to directly interact with acetyllysines [14]. Thus, the loss of these contacts in the *TAF1* variant p.Ser1600Gly may affect *TAF1* protein flexibility, potentially altering histone binding. The allele frequency from the ExAC database for the *TAF1* p.Ser1600Gly variant was zero; information about our variant was not found in Gnomad MAF. In addition, the z-score was 6.08, which implies increased constraint or that our *TAF1* variant is intolerant to variation. Collectively, these data suggest that the newly identified *TAF1* p.Ser1600Gly variant is rare, and mostly likely pathogenic.

### Neuronal ion channel gene expression analysis

Because abnormalities were observed in the brain of the proband, and sequencing analysis identified *TAF1* as a candidate variant, we next explored the effects of *TAF1*-depletion on neuronal ion channel gene expression since these genes have been associated with numerous inherited, and sporadic disorders of the brain and central nervous system [14]. SH-SY5Y cells are derived from human sympathetic neuronal tissue, and maintain many properties of nerve cells, thus providing a useful model for the characterization of molecules affecting human neuronal function, including endogenously expressed calcium channels [17–21]. In order to deplete *TAF1* expression *in vitro, TAF1*-specific siRNAs were transfected into SH-SY5Y cells, and depletion validated using qRT-PCR. Results showed that at the gene level, *TAF1* expression was decreased 44-fold (*P*-value <0.01). Using these *TAF1*-depleted cells, neuronal ion channel gene expression was determined using a focussed ion channel PCR array. Our results showed that five genes were up-regulated including acid sensing ion channel subunit 2 (*ASIC2*, MIM: 602866), acid sensing ion channel subunit 3 (*ASIC3*, MIM: 611741), potassium voltage-gated channel subfamily J member 14 (*KCNJ14*, MIM: 603953), calcium voltage-gated channel auxiliary subunit γ 4 (*CACNG4*, MIM: 606404), and potassium voltage-gated channel, shaker-related subfamily, β member 3 (*KCNAB3*, MIM: 604111) (Table 2). Three genes, calcium channel, voltage-dependent, T type, α 1G subunit (*CACNA1G*, MIM: 604065), hyperpolarization activated cyclic nucleotide-gated potassium channel 2 (*HCN2*, MIM: 602781), and potassium voltage-gated channel, subfamily H (eag-related), member 2 (*KCNH2*, MIM: 152427) were down-regulated (Table 2). The remaining 76 genes on the array showed no changes or were not detected (see Supplementary Table S1).

**Table 2:** Summary of neuronal ion channel gene expression in *TAF1*-depleted cells by qRT-PCR array analysis

Gene symbol	Gene description	Log 2 fold change
***ASIC2***	ASIC2 plays a role in neurotransmission	6.03
***ASIC3***	ASIC3 may play an important role in the detection of lasting pH changes	3.88
***KCNJ14***	KCNJ14 probably has a role in controlling the excitability of motor neurones	2.74
***CACNG4***	CACNG4 regulates both trafficking and channel gating of the AMPA receptors	2.00
***KCNAB3***	KCNAB3 regulates the activity of the α subunit	2.00
***CACNA1G***	CACNA1G subunit mediates the entry of calcium ions into excitable cells; also involved in a variety of calcium-dependent processes	−2.00
***HCN2***	HCN2 involved in the generation of native pacemaker activity in the heart and in the brain	−2.00
***KCNH2***	KCNH2 is a voltage-activated potassium channel belonging to the eag family	−2.21

Abbreviation: AMPA, alpha-amino-3-hydroxy-5-methyl-4-isoxazolepropionic acid receptor.

**Figure 4:**
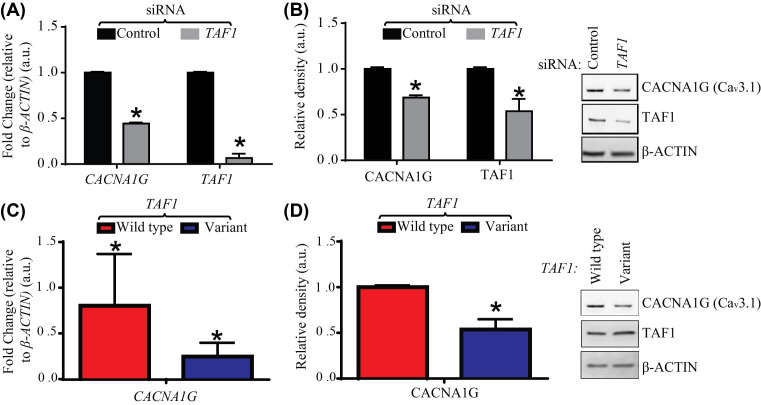
*TAF1*-depletion and transfection with *TAF1* variant, p.Ser1600Gly significantly alters significantly neuronal ion expression at both the gene and protein levels (**A**) SH-SY5Y cells were transfected with a commercially available *TAF1*-specific siRNA duplex or a scramble non-targetting control duplex. *β-ACTIN* (HKG), *TAF1*, and *CACNA1G* gene mRNA expression were analyzed using qRT-PCR. All gene expression were calculated relative to *B-ACTIN.* (**B**) Protein lysates were subjected to Western blot analysis with antibodies directed against TAF1, and CACNA1G; *β-*Actin served as a loading control. (**C**) SH-SY5Y cells were transfected with a *TAF1* wild-type plasmid expression vector or a *TAF1* variant p.Ser1600Gly plasmid expression vector. β-*ACTIN* (HKG), *TAF1*, and *CACNA1G* gene mRNA expression was analyzed using qRT-PCR. *TAF1* and *CACNA1G* gene expression of the *TAF1* variant p.Ser1600Gly was calculated respective to the expression of the *TAF1* wild-type plasmid samples. (**D**) SH-SY5Y cells were transfected with a TAF1 wild-type or TAF1 p.Ser1600Gly expression vector, protein isolated and subjected to Western blot analysis with antibodies directed against TAF1, CACNA1G, or *β-*Actin (loading control). For protein analysis, band intensities were quantitated from the 16-bit digital image by densitometry in ImageJ (NCBI) and are shown normalized to *β-*Actin control. All data are representative of three independent experiments with each sample repeated in triplicate.

We further evaluated *CACNA1G* because this gene was consistently down-regulated in both *TAF1*-depleted SH-SY5Y cells (Figure 4A,B) and IMR-32 cells (Supplementary Figure S3). In addition, *CACNA1G* is expressed in the cerebellum [22] and variants in the *CACNA1G* gene are associated with a hereditary cerebellar ataxia [23]. We observed altered neuronal ion channel gene expression in SH-SY5Y cells when we overexpressed *TAF1* with the *TAF1* variant p.Ser1600Gly plasmid expression vector. Figure 4C shows that when the *TAF1* variant p.Ser1600Gly is overexpressed, the expression of *CACNA1G* was down-regulated (four-fold change) (*P*-value <0.01). These results were also validated using immunoblotting (Figure 4D), where the protein level of CACNA1G (Ca_V_3.1) was also significantly down-regulated (approximately 46%) (*P*-value <0.01). Enforced expression of the *TAF1* variant p.Ser1600Gly had only a marginal effect on cell viability (Supplementary Figure S4). Taken together, these results show that abnormalities in the *TAF1* gene can result in alterations in gene expression.

### Calcium imaging

**Figure 5:**
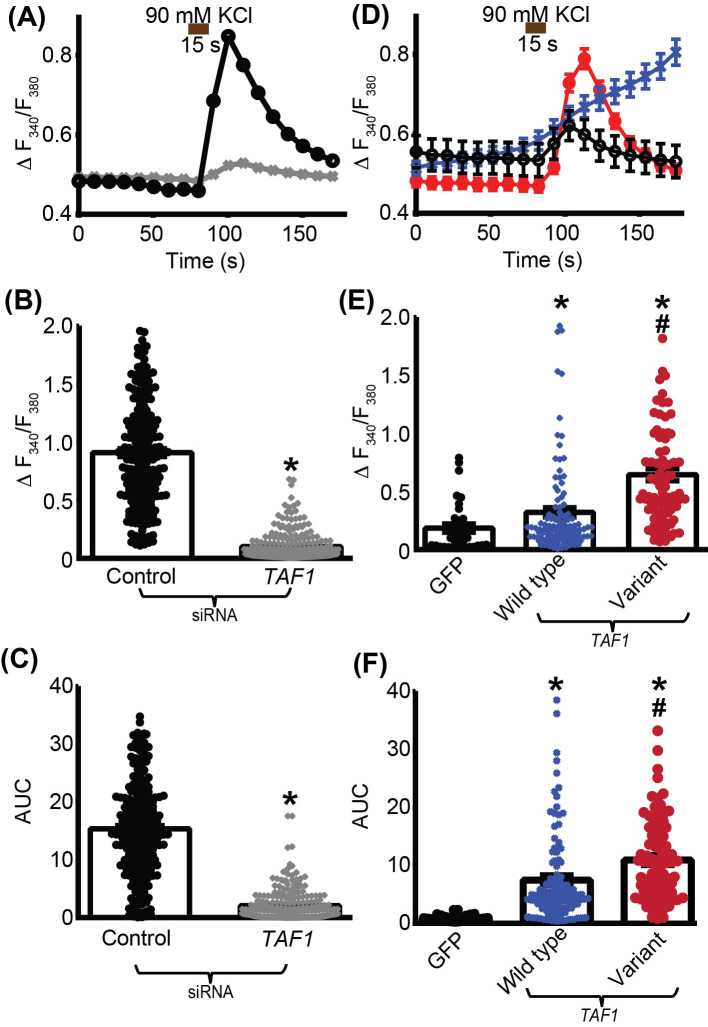
*TAF1* expression controls depolarization-evoked Ca^2+^ influx in SH-SY5Y cells Following a 1-min baseline measurement, cells were stimulated with 90 mM KCl for 15 s. (**A**) The average change in fluorescence ratio (F_340_/F_380_) over time for control-siRNA (open circles) or *TAF1*-siRNA (gray cross) transfected SH-SY5Y cells; most error bars are smaller than the symbols and cannot be seen here. Summary graph with scatter plot shows the (**B**) average peak fluorescence response (adjusted for background) or (**C**) AUC between 50 and 200 s for SH-SY5Y cells transfected as indicated. (**D**) The average change in fluorescence ratio (F_340_/F_380_) over time for GFP (open circles), *TAF1* wild-type (blue cross) or *TAF1* variant p.Ser1600Gly (red circles) transfected SH-SY5Y cells. Summary graph with scatter plot shows the (**E**) average peak fluorescence response (adjusted for background) or (**F**) AUC between 50 and 200 s for SH-SY5Y cells transfected as indicated. All data are representative of three independent experiments. B: *P<0.05 vs control (Student’s t-test); C: *P<0.05 vs control (Student’s t test); E: *P<0.05 vs GFP (One-way ANOVA with Tukey’s post hoc test ^#^*P*<0.05 vs wild type (One Way ANOVA with Tukey’s post hoc test) F: *P<0.05 vs GFP (One-way ANOVA with Tukey’s post hoc test; ^#^*P*<0.05 vs wild type (One-Way ANOVA with Tukey’s post-hoc test).

Calcium is a key signaling molecule and ion involved in a variety of diverse processes within the central nervous system [23]. Because manipulating *TAF1* expression (siRNA or overexpression) resulted in changes in *CACNA1G* gene expression, we examined whether calcium influx could be controlled by *TAF1* expression level. We first performed calcium imaging on SH-SY5Y cells where *TAF1* expression was depleted (siRNA). Calcium influx was evoked by depolarization using high 90 mM KCl solution perfused for 15 s. This elicited a peak calcium influx within 15–20 s of stimulation in control siRNA-transfected cells while *TAF1* siRNA-transfected cells failed to elicit a strong calcium influx (Figure 5A). Depleting *TAF1* expression resulted in a decreased peak calcium influx by 85% (*P*-value <0.0001, *n*=349-240 cells) (Figure 5B) and decreased the area under the curve (AUC) by 89% (*P*-value <0.0001, *n*=349-240 cells) (Figure 5C) compared with control siRNA transfected cells. We next asked if increasing *TAF1* expression levels would affect depolarization evoked calcium influx and if expression of the *TAF1* variant p.Ser1600Gly would alter this function. SH-SY5Y cells were transfected using either *TAF1* wild-type, *TAF1* variant p.Ser1600Gly, or the corresponding empty control plasmid. This plasmid allows for the expression of a GFP under the dependence of an IRES sequence. Only the transfected cells (identified by GFP fluorescence) were analyzed. Depolarization elicited a peak calcium influx within 15–20 s of stimulation in all conditions (Figure 5D). *TAF1* wild-type transfected cells do not show a rebound after the calcium influx but a steady increase instead (Figure 5D). Overexpressing *TAF1* wild-type or *TAF1* variant p.Ser1600Gly resulted in an increased peak calcium influx by 77 and 256%, respectively (*P*-value <0.0001, *n*=36–118 cells) (Figure 5E), compared with empty control plasmid transfected cells. This effect was correlated with an increased AUC, which was 10 times (for *TAF1* wild-type) and 15 times (for *TAF1* variant p.Ser1600Gly) the value of the empty control plasmid transfected cells (*P*-value <0.0001, *n*=36–118 cells) (Figure 5F) compared with control siRNA transfected cells. These results show a role for *TAF1* into regulating the activity of T-type voltage gated calcium channels.

### Neurite outgrowth

**Figure 6:**
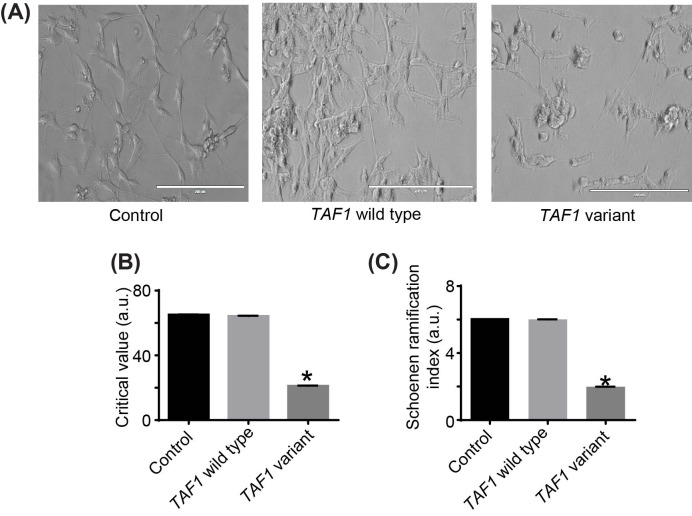
The *TAF1* variant p.Ser1600Gly significantly effects differentiation of SH-SY5Y cells (**A**) Morphology of empty vector (control), TAF1 wild-type, and TAF1 p.Ser1600Gly transfected SH-SY5Y cells; 20× magnification; scale bar is approximate. (**B**) The critical values for both the control and wild-type are similar (65.27 compared with 64.43), while the value for the variant is 21.29, significantly different [F (2, 6) = 53469, *P*<0.01]. (**C**) For the SRI, this value is also significantly different from both the control and wild-type conditions [F (2, 6) = 1.5 × 107, *P*-value <0.01]. The data are expressed as mean ± S.D. of three independent experiments performed in triplicate.

Neurite outgrowth is the process whereby neural cells extend new projections as they respond to guidance cues, reviewed in [24]. During development, neurite outgrowth contributes to the formation of the functional nervous system and the brain [25]. Due to the abnormalities observed in the proband’s MRI, we sought to elucidate whether or not the *TAF1* variant p.Ser1600Gly had any effect on neurite outgrowth of SH-SY5Y cells. To do this, we overexpressed the *TAF1* variant p.Ser1600Gly plasmid expression vector, added differentiation medium for 7 days, and analyzed neurite formation. Results showed that there was a statistically significant difference between *TAF1* wild-type and *TAF1* variant p.Ser1600Gly as determined by one-way ANOVA for all key metrics of the Sholl analysis (Figure 6A). The critical value represents the maximum number of process crossings, and is closely related to the process maximum. As the results show, the critical values for both the control and wild-type are similar (65.27 compared with 64.43), while the value for the variant is 21.29, significantly different [F (2, 6) = 53469, *P*-value <0.01] (Figure 6B). Another metric, the Schoenen ramification index (SRI) is the ratio between number of branches at the maximum and the number of primary branches. Comparing groups, this value is also significantly different from both the control and *TAF1* wild type [F (2, 6) = 1.5 × 10^7^, *P*-value <0.01] (Figure 6C). We assessed cell viability by Trypan Blue exclusion and we found no marked differences in cell viability between the empty vector control and wild-type (Supplementary Figure S4). These data suggest that the *TAF1* p.Ser1600Gly variant impairs neuronal process differentiation.

### Cell proliferation

**Figure 7:**
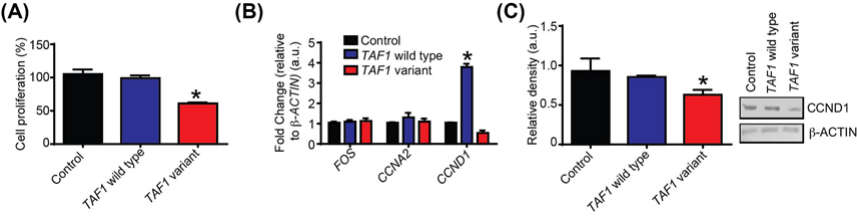
Transfection with *TAF1* variant p.Ser1600Gly significantly alters cell proliferation and cyclin expression at both the gene and protein levels (**A**) SH-SY5Y cells were transfected with either an empty vector control, a *TAF1* wild-type plasmid expression vector or *TAF1* variant p.Ser1600Gly plasmid expression vector. A cell proliferation assay kit was used for measurement of cell proliferation 48 h after the transfection. The percentage of cell proliferation in each sample was calculated respective to the absorbance of empty vector control samples. (**B**) SH-SY5Y cells were transfected with either an empty vector control, *TAF1* wild-type plasmid expression vector or *TAF1* variant p.Ser1600Gly plasmid expression vector. *β-*Actin (HKG), *FOS* (MIM: 164810), *CCNA2* (MIM: 123835), and *CCND1* mRNA expression were analyzed using qRT-PCR. Gene expression of the *TAF1* variant p.Ser1600Gly was calculated relative to *β-*Actin. (**C**) Protein lysates were subjected to Western blot analysis with antibody directed against Cyclin-D1; *β-*Actin served as a loading control. Band intensities were quantitated from the 16-bit digital image by densitometry in ImageJ (NCBI) and are shown normalized to *β-*Actin. All data were representative of three experiments with each sample repeated in triplicate.

As gene expression was altered by *TAF1* depletion, we next asked if cell proliferation was also affected. Cell proliferation involves the co-ordinated expression of protein encoding genes that control progression through the cell cycle. These regulators include *CCND1* (MIM: 168461), a growth factor sensor that integrates extracellular signals with the core cell cycle machinery [26]. In addition, previous studies clearly show that expression of *CCND1* is transcriptional regulated by *TAF1* [27,28]. We investigated the effects of the variant *TAF1* p.Ser1600Gly on cell proliferation, and *CCND1* expression. We found that overexpression of the variant *TAF1* p.Ser1600Gly had an effect on cell cycle proliferation. At 48 h post-transfection, the percentage of cell proliferation in the variant samples (61%) were significantly different when compared with the empty vector control (100%) and wild-type (99%) (*P*-value =0.01). In addition, the *TAF1* p.Ser1600Gly variant decreased *CCND1* expression at the gene (1.8-fold) (*P*-value =0.018) and protein levels (62%) (*P*-value =0.01) (Figure 7). Similar findings were observed in Cathecholamine-A-differentiated (CAD) and IMR-32 cells (Supplementary Figure S5). The p.Ser1600Gly variant also decreased *CCNA2* gene expression (Supplementary Figure S5B,C).

## Discussion

Unbiased genome-wide molecular tools such as NGS have a proven efficacy, although they produce genetic and genomic information that can be difficult to interpret. Here, we used WGS and RNA sequencing (RNA-seq) analysis to identify a novel variant in *TAF1* in a patient with global developmental delay (motor, cognitive, and speech), hypotonia, possibly ataxia, and cerebellar hypoplasia of unknown origin. Together with other studies [7,8], the present study illustrates the capability to identify disease-causing gene variants using an integrative genomic approach.

X-inactivation (XCI, also called lyonization) is an epigenetic, gene dosage compensatory mechanism that occurs by inactivation of copy of the X-chromosome in cells. Random XCI of one of the parental chromosomes results in an approximately equal proportion of cells expressing alleles from either the maternally or paternally inherited active X, and is defined by the XCI ratio [29]. A skewed XCI ratio (i.e. 90:10) is suggestive of non-random inactivation, which can play an important role in X-linked genetic conditions. We observed an X-inactivation ratio of 96:4 in the mother of the proband, which is consistent with a highly skewed X-inactivation pattern (Supplementary Figure S1). These data are in agreement with the previous findings of O’Rawe et al. [4], who also identified two female carriers of *TAF1* gene defects with highly skewed XCI.

**Figure 8:**
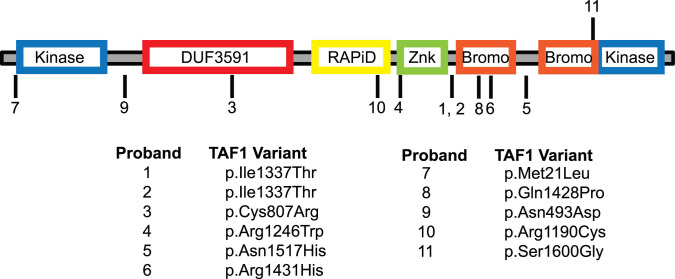
Summary of the damaging, potentially disease associated variants of TAF1 The TAF1 variants are indicated in black. The domains of TAF1 are illustrated and annotated in the diagram. *TAF1* variants in probands (1–6) were previously reported by O’Rawe et al. [4]. *TAF1* variants in proband 7 and 8 were reported by Niranjan et al. [15]. The *TAF1* variants probands 9 and 10 were reported by Hu et al. [16]. The *TAF1* variant in proband 11 was reported by the present study. *TAF1* gene sequence NM_004606 was used for alignment of the data.

TAF1 is a unique protein, in that it has been reported to possess intrinsic protein kinase activity [28,30–32], a DU3591 domain with histone acetyltransferase activity [27,33], a zinc knuckle domain [34], and two tandem bromodomains [14,33–36]. Due to its large size and solubility issues, structural information for TAF1 is limited [36,37]. Hence, the structural basis for the kinase and HAT activity of TAF1 remains elusive. Nevertheless, pathogenic variants within the *TAF1* gene have been identified in several putative protein domains [3,29,30] (Figure 8). Our study is the first to report a variant within the second bromodomain of TAF1. Since the function of the bromodomain is to recognize acetylated lysine residue on histones, alterations in DNA that modify amino acid residues will mostly result in aberrant histone binding, and consequently changes in gene expression. We decided to perform focussed gene array analysis to identify genes that might be altered by *TAF1* variant. By doing so, we observed differential gene expression in several neuronal ion channels in both *TAF1*-depleted cells and those transfected with the *TAF1* variant p.Ser1600Gly plasmid expression vector. Interestingly, we observed that disrupting *TAF1* (i.e. siRNA silencing or overexpression of the p.Ser1600Gly variant) increased the expression of some genes (i.e*. ASIC2* and *ASIC3*) while others were suppressed (i.e. *CACNA1G* and *HCN2*). These data are in agreement with previous findings using *TAF1*-depleted *Drosophila* cells, which showed that *TAF1* regulates the amplitude of the transcription burst and fine tunes transcription [38]. Thus, loss of function of *TAF1* can positively or negatively deregulate gene expression. Our data suggest that the newly identified *TAF1* p.Ser1600Gly variant leads to a reprogramming of neuronal gene expression that culminates in altered membrane excitability, proliferation, and differentiation.

There is some evidence that *TAF1* plays a functional role in neurones [39,40], however, the data are limited. Results of Sholl analysis showed our *TAF1* variant p.Ser1600Gly decreases the length of dendrites and the number of interactions (Figure 6). The development and patterning of dendrites is a tightly regulated process that is essential for proper functioning of the central nervous system [34] as dendrites receive afferent input [35]. As such, their form determines the regions with which a neurone can communicate, and how synaptic signals are integrated. Therefore, the branching of a neurone’s dendrites dictates its function [35]. As the branches here are shorter, local neural circuitry may be affected. This may partially explain why individuals affected with *TAF1-*ID syndrome have brain anomalies.

In the present study, we noted a 4× increase in *CCD1* gene expression in cells transfected with a wild-type TAF1 vector, however cyclin D1 protein levels were not significantly different. This discrepancy in CCD1 gene expression and cyclin D1 protein levels might be due to translation efficacy being rate limiting. Further studies are required to address this issue. We also observed a decrease in cell proliferation, which was associated with lower *CCND1* gene and protein levels. These data are consistent with previous studies carried out in the ts13 variant hamster cell line. The ts13 variant hamster cell line contains a temperature-sensitive missense variant in the DUF3591domain of *TAF1* which causes G_1_/S phase cell cycle arrest and transcriptional down-regulation of *CCND1* but not *FOS* (MIM: 164810) [41,42]. Additionally, loss of *CCND1* reportedly impairs cerebellar development [43].We suggest that attenuation of cell proliferation by transcriptional down-regulation of *CCND1* may play a critical role in brain anomalies associated with *TAF1*-ID syndrome.

In conclusion, our study resolves an undiagnosed case of global developmental delay (motor, cognitive, and speech), hypotonia, possibly ataxia, and cerebellar hypoplasia of unknown origin as we found a new variant in the *TAF1* gene, p.Ser1600Gly. These results add another example of X-linked *TAF1*-ID syndrome (MIM: 300966). Our experiments demonstrate dramatic effects of the *TAF1* variant p.Ser1600Gly on gene expression. Many of these genes are required for normal brain function and development. As such, further investigation of this rare genetic disease is greatly needed, and the development of an animal of *TAF1*-ID syndrome is warranted.

## Consent to publish

A written informed consent for publication of clinical and medical images was obtained from the responsible family members of the patient.

## Availability of data and materials

The datasets developed, used, and/or analyzed during the study have been submitted to dbGAP, but the data download is not active yet.

## Supplementary Material

Supplementary Tables

supplementary Figures

## Abbreviations

ASIC2: acid sensing ion channel subunit 2
ASIC3: acid sensing ion channel subunit 3
AUC: area under the curve
CACNA1G: calcium channel, voltage-dependent, T type, α 1G subunit
CSF: cerebrospinal fluid
CT: computer tomography
EEG: electroencephalogram
ExAC: Exome Aggregation Consortium
GAPDH: glyceraldehyde-3-phosphate-dehydrogenase
GOI: gene of interest
HCN2: hyperpolarization activated cyclic nucleotide-gated potassium channel 2
HKG: housekeeping gene
IGF-1: insulin growth factor-1
IRES: internal ribosome entry site
NGF: never growth factor
NGS: next-generation sequencing
qRT-PCR: quantitative real-time PCR
RA: retionic acid
RNA-seq: RNA sequencing
TAF: TATA box-binding protein associated factor
TAF1: TATA-box-binding protein factor 1
TAF1-ID: *TAF1* intellectual disability
TBP: TATA box-binding protein
TFIID: transcription factor II D
TGen: The Translational Genomics Research Institute
VUS: variant of unknown significance
WGS: whole genome sequencing
XCI: X-chromosome inactivation
