# Supplementary material for: A novel variant in TAF1 affects gene expression and is associated with X-linked TAF1 intellectual disability syndrome
Source: Neuronal Signal. 2018 Jul 16;2(3):NS20180141. doi: 10.1042/NS20180141 (PMC7373232; doi:10.1042/NS20180141)
Supplement: Supplementary Tables [file ns-02-ns20180141_supp.zip › ns-02-ns20180141_supp2.pdf]

Supplemental Methods and Materials

Cell Culture

IMR-32 cells were grown in modified Eagle medium (MEM) (Gibco, Gaithersburg, MD) supplemented with 10% fetal bovine serum (FBS) (HyClone, South Logan, UT), 2 mM l-glutamine, and penicillin-streptomycin (HyClone), 100mM sodium pyruvate (Corning, Corning, NY), and 100mM non-essential amino acids (Caisson, Smithfield, UT) in a humidified incubator containing 5% CO2 at 37°C. CAD cells were grown in Dulbecco's Modified Eagle Medium: Nutrient Mixture F-12 (DMEM/F-12) (Gibco) media containing 1% non-essential amino acids, 1% sodium pyruvate, and 1% penicillin/streptomycin supplemented with 10% FBS, in a 95% air/5% CO2 humidified incubator at 37ºC. All cells were grown to confluency (80%) and the same passage number was used for all experiments.

Cell Viability Assay

Briefly, all cells were treated according to the manufacturer’s instructions using siTrans; the concentration of DNA used was 360 ng. Complete growth media was added after 6 hours to ensure cell health. The samples were then collected at 24h using 0.05% trypsin, pelleted, and re-suspended for counting. For live/dead cell counting, 10 uL of the cell suspension was mixed with an equal volume of trypan blue, and counted using an automated cell counter (Life Technologies Countess™ II Automated Cell Counter).

Whole Exome Sequencing

Whole exome sequencing (WES) from the blood of the **maternal grandfather** was performed at TGen. Exomic libraries were prepared with the SureSelect V5+UTR capture kit (Agilent Technologies, Santa Clara, CA), following the manufacturer’s protocol. Sequencing was

performed by 101 bp paired-ends sequencing on a HiSeq2000 instrument (Illumina, San Diego, CA). For analysis at TGen, filtered reads were aligned to the Human genome (Hg19/GRC37) using the Burrows-Wheeler transform (BWA v.0.79.) [42]. PCR duplicates were removed using Picard v1.92 [43] and base quality recalibration. Indel re-alignment and SNP and indel discovery were performed using the Genome Analysis Toolkit (GATK v2.5-2) [43]. Data was filtered against dbSNP137, 1000 Genomes, dbSNP3.2, and then annotated with SnpEff 3.2a against Ensembl v66 to identify novel pathogenic changes. After filtering for putative functional variants with a population minor allele frequency below 1% . Only 1 variant (MECP2 VUS) fit Mendelian model. The MECP2 variant was ruled out due to reports showing benign, clinical experience, the child's phenotype, and after examination of literature showing the MECP2 variant identified in a child that was labelled male was heterozygous and likely not male [44]. Taken together with our previous WGS sequence analysis suggested that TAF1 as the sole candidate gene.

Phased Allele Expression Analysis

The RNA sequencing data from the mother and the affected proband as well as whole exome sequencing data from the grandfather were subjected to phased allele expression analysis as previously described [27]. Briefly, bases with Phred quality score > 20 were utilized in the analysis. Pile-up was parsed by an in-house perl script. Next, the allelic ratio at each heterozygous locus was calculated by dividing the number of reads mapping to the variant allele by the total number of reads mapping to the locus. After allelic ratio calculation, the SNPs were further filtered for quality. SNPs within the PAR1 and PAR2 pseudo-autosomal regions were filtered out as they follow autosomal inheritance and can bias XCI ratio[45]. They were then

filtered for high confidence variant loci from exome dataset with a genotype filter score of PASS by GATK Variant Recalibrator[43]. Lastly, both loci without a dbSNP identifier and variants with less than 20X coverage were filtered out. Phasing of X-linked heterozygous variants allows us to evaluate the functional profile of each inherited parental copy. By estimating the parameters (mean, variance) of each copy's allele ratio distribution, we can estimate the proportion of cells with Xm or Xp as active and inactive (e.g., mean allelic ratio of paternal alleles of 65 and mean allelic ratio of maternal alleles of 35 equals an estimated XCI ratio of 65:35). To control for over-dispersion of read count data from RNA- seq, phased allelic ratios were fitted to the beta distribution to estimate their mean and variance using the fitdistr module of MASS package in R[46-48].

References:

27. Szelinger S, Malenica I, Corneveaux JJ, Siniard AL, Kurdoglu AA, Ramsey KM, Schrauwen I, Trent JM, Narayanan V, Huentelman MJ *et al*: **Characterization of X Chromosome Inactivation Using**

42. Li H, Durbin R: **Fast and accurate short read alignment with Burrows-Wheeler transform**. *Bioinformatics* 2009, **25**(14):1754-1760.

43. McKenna A, Hanna M, Banks E, Sivachenko A, Cibulskis K, Kernytsky A, Garimella K, Altshuler D, Gabriel S, Daly M *et al*: **The Genome Analysis Toolkit: A MapReduce framework for analyzing next-generation DNA sequencing data**. *Genome Research* 2010, **20**(9):1297-1303.

44. Kim IJ, Kim YJ, Son BH, Nam SO, Kang HC, Kim HD, Yoo MA, Choi OH, Kim CM: **Diagnostic mutational analysis of MECP2 in Korean patients with Rett syndrome**. *Exp Mol Med* 2006, **38**(2):119-125.

45. Mangs AH, Speirs HJL, Goy C, Adams DJ, Markus MA, Morris BJ: **XE7: A novel splicing factor that interacts with ASF/SF2 and ZNF265**. *Nucleic Acids Research* 2006, **34**(17):4976-4986.

46. Skelly DA, Johansson M, Madeoy J, Wakefield J, Akey JM: **A powerful and flexible statistical framework for testing hypotheses of allele-specific gene expression from RNA-seq data**. *Genome Research* 2011, **21**(10):1728-1737.

47. Zhou YH, Xia K, Wright FA: **A powerful and flexible approach to the analysis of RNA sequence count data**. *Bioinformatics* 2011, **27**(19):2672-2678.

48. Hardcastle TJ, Kelly KA: **baySeq: empirical Bayesian methods for identifying differential expression in sequence count data**. *BMC Bioinformatics* 2010, **11**:422.

Table S1. Summary of whole genome sequencing statistics.

	Proband	*Father*	*Mother*
WGS Reads Generated	1.05E+09	1.14E+09	1.17E+09

WGS Reads Aligned	1.03E+09	1.14E+09	1.14E+09
WGS Duplicate Rate	16.7	13.00%	14.50%
WGS Mean Base Coverage	26.6	30.7	30.2
mRNA Reads Generated	7.00E+07	8.57E+07	9.08E+07

Table S2. Sequence of specific gene primers used for qRT-PCR

**Gene**	**Sequence**
GAPDH	5'- AATGGGCAGCCGTTAGGAAA-3'
	3'- GCGCCCAATACGACCAAATC -5'
ACTIN	5'- CTCGCCTTTGCCGATCC -3'
	3'- GGGGTACTTCAGGGTGAGGA -5'
TAF1	5'- AAACACTCCGCGAAAACGTG -3'
	3'- TAGGGCCCTGGGGTCATTG-5'

CACNA1G	5'- AGAAGGAAAGCCCAGTGCAA -3'
	3'- CGGAAACCAAAGGCCACAAG -5'
FOS	5`-CAGACTACGAGGCGTCATCC- 3`
	3`-GTTCGCCTCTGTCTGGTTGA-5'
CCNA2	5`-ACCCAGGGTTCTCAGAATGG- 3`
	3`-ACTCATTCTGACCGTAGGTTC-5'
CCND1	5`-CTGATTGGACAGGCATGGGT-3`
	3`-ATGGCAACTGAAGGTCCGTG-5'

Table S3. List of genes encoding for different subunits of the ion channels.

See attached excel file.
