# Supplementary material for: A novel variant in TAF1 affects gene expression and is associated with X-linked TAF1 intellectual disability syndrome
Source: Neuronal Signal. 2018 Jul 16;2(3):NS20180141. doi: 10.1042/NS20180141 (PMC7373232; doi:10.1042/NS20180141)
Supplement: supplementary Figures [file ns-02-ns20180141_supp1.pdf]

Supplemental Figure Legends

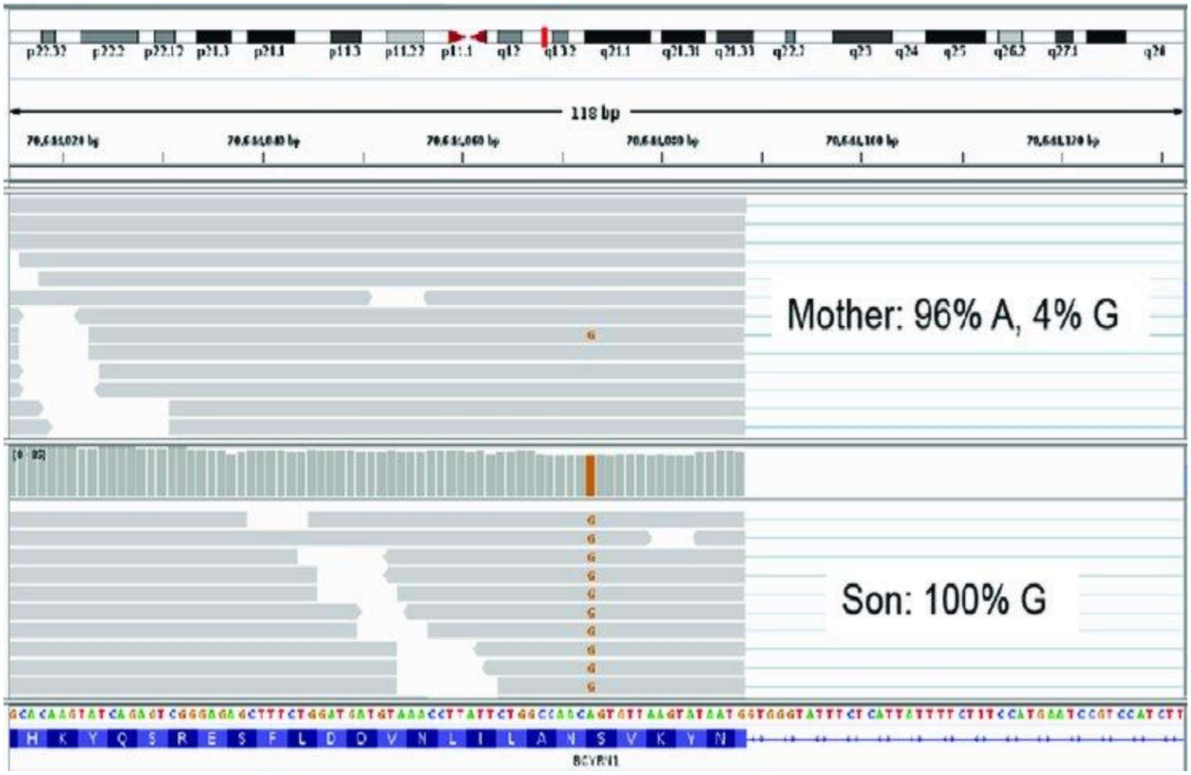

Figure S1. RNA-sequencing analysis showing X-inactivation. A.) Unaffected mother has strong XCI skew expressing *TAF1* variant c.4735A>G, p.Ser1600Gly unlike the affected proband. B.) Model depicting rare recombination event identified in the present study.

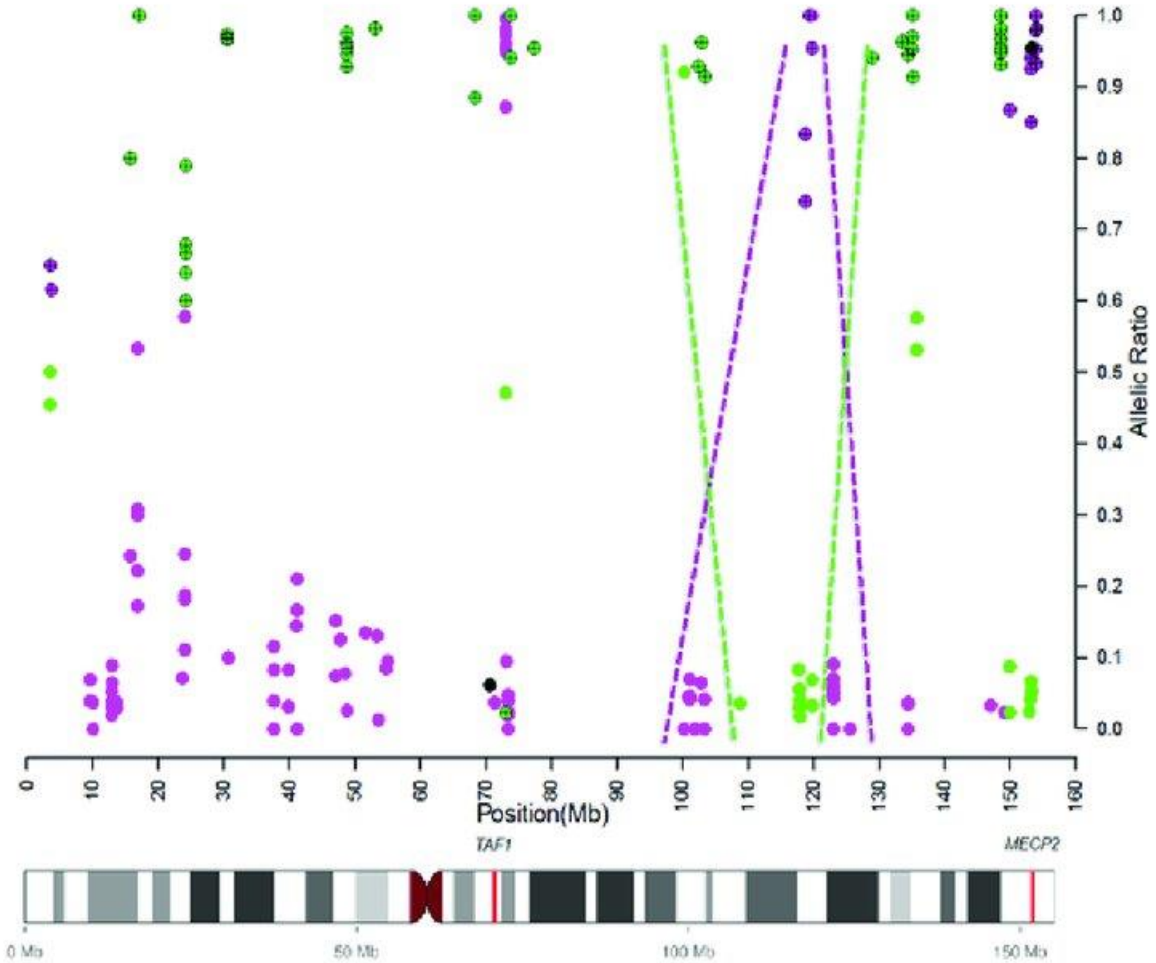

Figure S2. Phased allelic expression of gene variants on the X-chromosome. This plot indicates the expression of X-chromosome variant alleles identified using RNA sequencing data from the proband and the mother and WGS data from the grandfather. The x-axis indicates chromosomal position along X-chromosome, while the y-axis indicates the allelic ration of the variant allele; the total number of variants plotted was 175. The magenta dots indicate the allelic ratio of variant alleles that are passed on to the proband thus located on the same maternal X. The green dots indicate variant allele ratios for variants that are located on the other copy of X, which are not passed on to the proband. The black dots indicate variant allele ratios for the TAF1 and MECP2 genes on the X-chromosome. The plot shows the variant transmitted from the grandfather. In addition, the plot shows that there was no recombination from grandfather to

mother, and mother is almost entirely expressing grandfather's X. Note the fraction of 0.74 for grandfather's X in mother, except for a 15 Mb section that has undergone recombination. The dotted lines show that there are two recombination spots from the mother to the child.

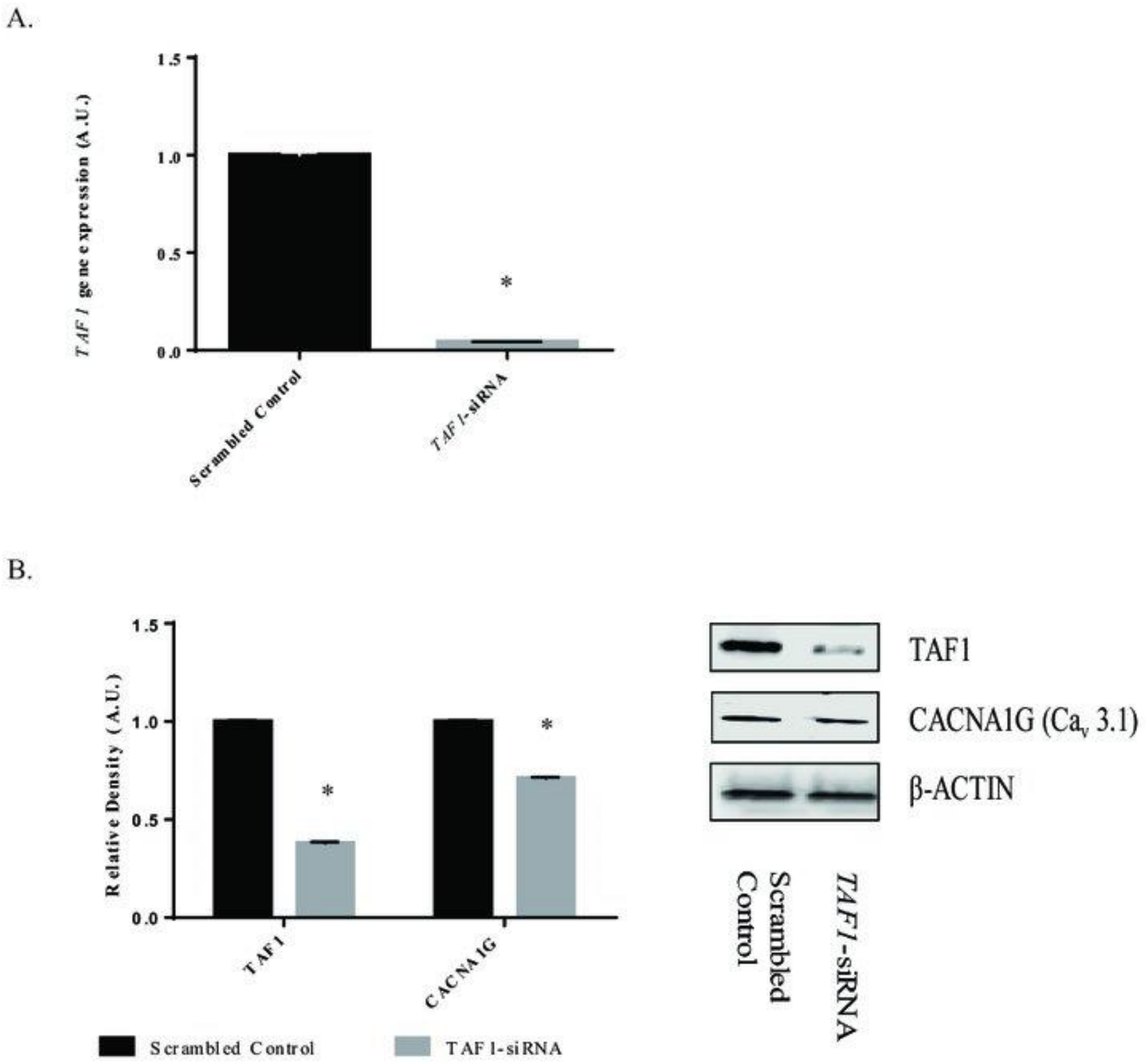

Figure S3. ***TAF1*-depletion significantly alters neuronal ion channel expression at both the gene and protein levels.** A.) IMR-32 cells were transfected with a commercially available *TAF1*-specific siRNA duplex, and a scrambled non-targeting control duplex. β-*ACTIN* (housekeeping gene), *TAF1*, *CACNG4*, and *CACNA1G* mRNA expression was analyzed using

qRT-PCR. All gene expression was calculated respective to the expression of the matched non-targeting scrambled control samples. B.) Protein lysates were subjected to Western blot analysis with antibodies directed against TAF1, CACNG4, and CACNA1G; β-ACTIN served as a loading control. For protein analysis, band intensities were quantified from the 16-bit digital image by densitometry in Image J (NCBI) and are shown normalized to the respective control. All data are representative of three independent experiments with each sample repeated in triplicate.

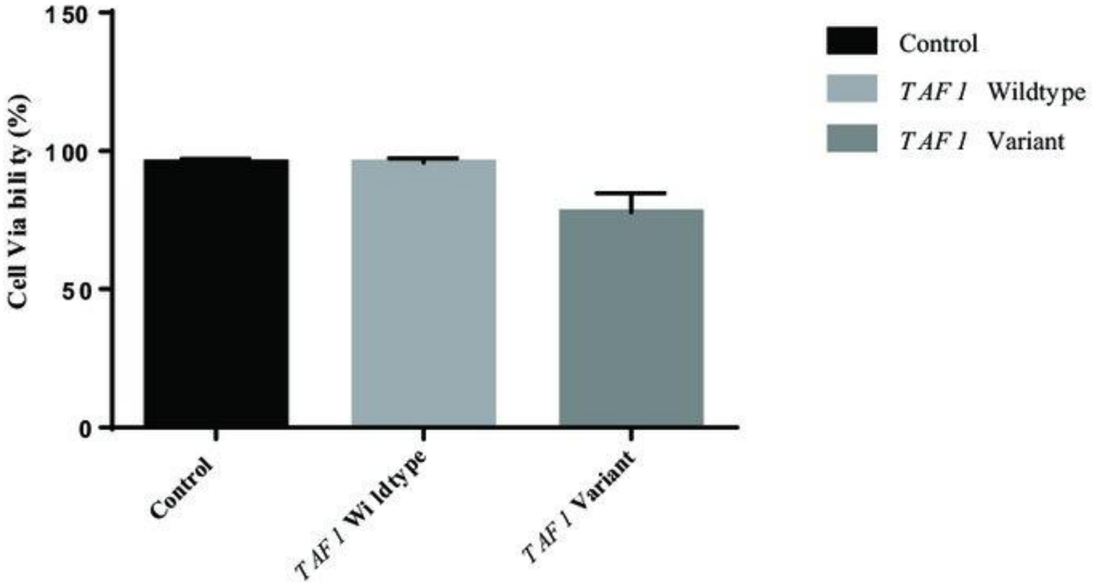

Figure S4. The effect of the *TAF1* variant p.Ser1600Gly on the viability of SH-SY5Y cells. Briefly, all cells were treated according to the manufacturer's instructions using siTrans; complete growth media was added after 6 hours to ensure cell health. The samples were then collected at 24h using 0.05% trypsin, pelleted, and re-suspended for counting. For live/dead cell counting, 10 uL of the cell suspension was mixed with an equal volume of trypan blue, and counted. There was a significant difference between the groups using the Student's t-test (p-

value = 0.03). The data are expressed as mean ± standard deviation (SD) of three independent experiments performed in triplicate.

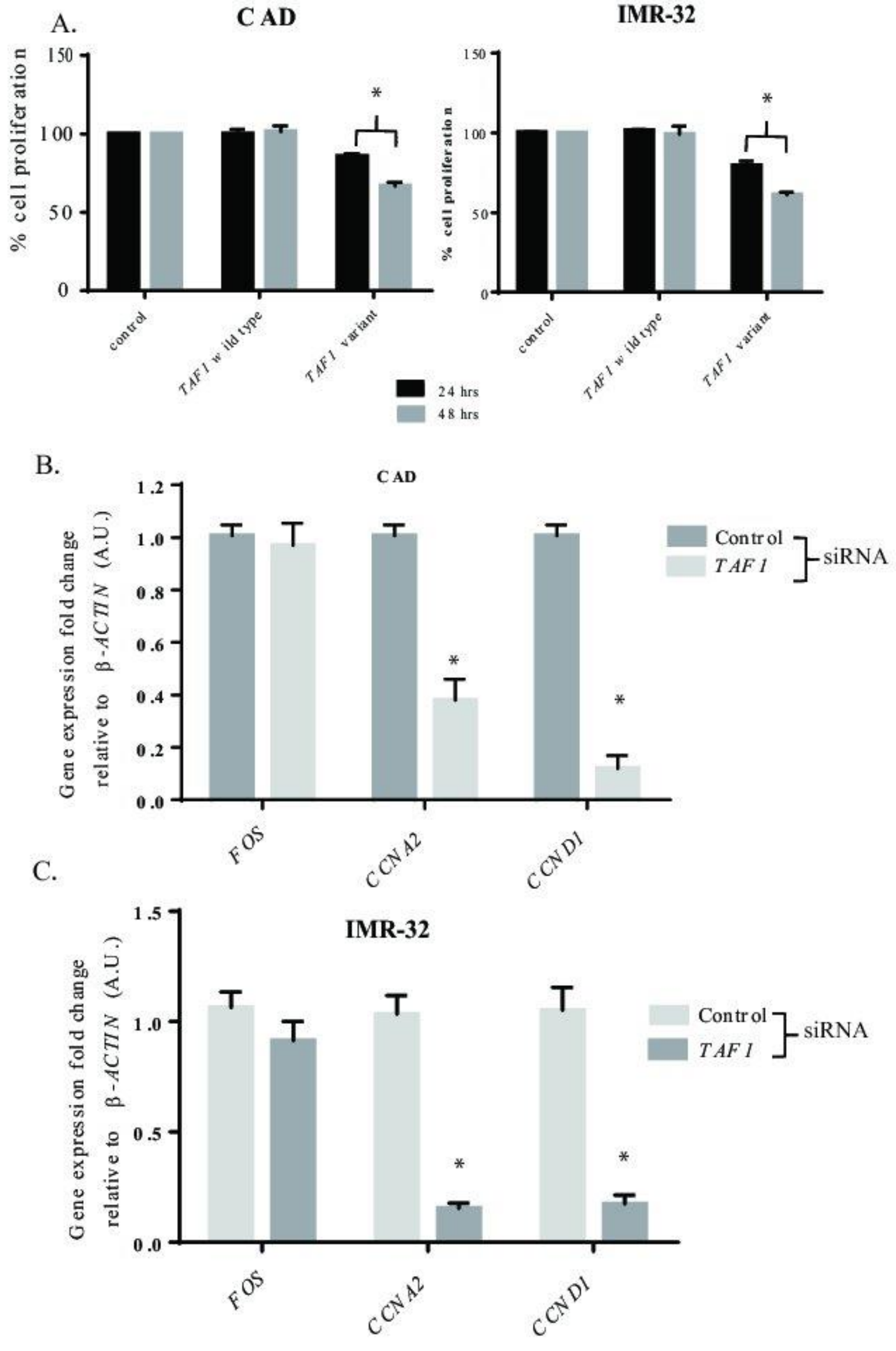

Figure S5. *TAF1*-depletion and transfection with *TAF1* variant p.Ser1600Gly alters cell proliferation and cyclin expression at both the gene and protein levels. A.) CAD and IMR-32

cells were transfected with either an empty vector control, a *TAF1* wild type plasmid expression vector, or a *TAF1* variant p.Ser1600Gly plasmid expression vector. A cell proliferation assay kit (Quick Cell Proliferation Assay Kit, Abcam, Cambridge, MA) was used for measurement of cell proliferation 24h and 48 h after the transfection. The percentage of cell proliferation in each sample was calculated respective to the absorbance of empty vector control samples. CAD (B.) or IMR-32 cells (C.) were transfected with a commercially available *TAF1*-specific siRNA duplex, and a scrambled non-targeting control duplex. *β-ACTIN* (housekeeping gene), *FOS*, *CCNA2*, and *CCND1* gene mRNA expression were analyzed using qRT-PCR. *FOS*, *CCNA2*, and *CCND1* gene expression were calculated relative to *β-ACTIN*. All data represents three independent experiments with each sample repeated in triplicate.
